# Comparing traditional surveys and web-scraped data to understand the pigeon racing industry in Southern California

**DOI:** 10.3389/fvets.2024.1451198

**Published:** 2025-02-03

**Authors:** Maurice Pitesky, Malekah Isa, Charlene Rivera, Elise Streba, Joseph D. Gendreau

**Affiliations:** ^1^School of Veterinary Medicine, University of California, Davis, Davis, CA, United States; ^2^Animal Health Branch, California Department of Food and Agriculture, CA, United States; ^3^Veterinary Services, Animal and Plant Health Inspection Service (USDA), Ames, IA, United States

**Keywords:** web-scraping, social media, surveys, pigeons, virulent Newcastle Disease

## Abstract

Recent outbreaks of virulent Newcastle disease (vND) in domestic poultry in Southern California have raised questions as to the source(s) and the mode(s) of transmission. While pigeons have been found to be capable of transmitting a variant of Newcastle disease virus (NDV) known as Pigeon paramyxovirus-1 (PPMV-1) to other avian species including poultry, the potential geographic distribution and scope of transmission from racing pigeons to other domestic poultry in Southern California is poorly understood. In order to better understand the potential risk of transmission of vND from racing pigeons to domestic poultry, an extension-initiated survey was conducted in addition to social-media based analysis of racing pigeon activity associated with postings on a web-based popular classified advertisement (CAW) in Southern California. Likely due to the insularity of the pigeon racing industry in Southern California, only 1 survey response was completed. However, monitoring results from the CAW demonstrated a much higher level of social media activity associated with racing pigeon sales in Southern California. Specifically, 191 unique posts associated with pigeon sales were flagged between 12/21/2020 and 1/21/2021 demonstrating the potential for the analysis of social media as an extension-based tool. Data from the CAW showed that the majority of pigeon related posts and sales came from both the Inland Empire region of Southern California and San Diego county which overlapped with the established vND control zone. From an extension perspective, while traditional-based surveys can still provide valuable information, web-scraping of social media can provide a continuous source of spatio-temporal data that can facilitate extension-based observations and analysis among hard to reach stakeholder groups.

## Introduction

Virulent Newcastle disease is caused by avian orthoavulavirus serotype 1 that is transmitted by inhalation or ingestion of the virus from respiratory secretions and feces of infected birds (1). While the disease is zoonotic and can potentially cause mild conjunctivitis in humans, in birds vND is highly contagious and fatal (2). Because Newcastle disease is thought to infect all avian species, the disease can be spread between domestic and wildlife and vice versa (2). For example, with respect to pigeons, a pigeon variant of NDV, Pigeon paramyxovirus-1 (PPMV-1) has been found to be capable of spreading to domestic birds including poultry (3).

Between 2018 and 2020, an outbreak of vND in commercial and non-commercial poultry affected approximately 476 premises primarily in Southern California (4). At this point the primary mode of transmission was thought to be a combination of poor biosecurity and exposure to the virus from backyard and gamefowl (i.e., fighting birds). However, other potential transmission routes, including transmission from wild to domestic birds and domestic to domestic transmission (e.g., racing pigeons to commercial poultry), are poorly understood in Southern California. Since the literature supports historical PMV-1 infection in racing pigeons in California (4, 5) and the possibility of transmission of vND from wild pigeons to poultry (6), developing a better understanding of the risk of transmission between racing pigeons and domestic poultry in Southern California is an important aspect of potential disease transmission to understand. Unfortunately, gaining knowledge about the racing industry through traditional extension-based methods including participation in surveys is challenging due to the insular nature of the racing pigeon industry. The ability of pigeons to fly hundreds of kilometers and return to their home lofts has been encapsulated into the sport of pigeon racing (7, 8). In California, pigeon racing typically includes around 200–1,000 pigeons per race where all racing pigeons are marked and driven 240–965 miles away to be released to start the race. Each pigeon’s final destination is different depending on where the pigeon lives. Due to the spatial distribution of the pigeons during these races, the potential for disease transmission exists. Therefore, a better understanding of the basic practices associated with the racing pigeon industry in Southern California is necessary to better understand the potential risk of vND transmission in Southern California.

In order to better understand these risks two approaches were explored. First a traditional survey was developed that focused on several relevant topics associated with the pigeon racing industry in.

Southern California with a focus on spatio-temporal distribution during races, trade of pigeons, behavior of pigeons during races, and biosecurity. In parallel, a supplementary approach using publicly available online data was developed to approximate the geographic distribution and relative prevalence of racing pigeon ownership. Specifically, a classified advertisement website (CAW) was identified as a significant marketplace for pigeons and other domestic birds in California.

For background purposes, web-scraping (i.e., data extraction) from social media and other internet-based data sources represent a new method of analyses for veterinary extension purposes. In short web-scraping is the process of programmatically extracting selected data from specific websites (9, 10) The web-scraped data can be further analyzed via various methods including geographic information (11), topic modeling (12), and sentiment analysis (13) to understand various types of public data in a continuous fashion (14). While this approach is commonly used in the human social sciences with examples ranging from sentiment associated with vaccines (15) and infectious disease monitoring (10, 16), the potential for similar applications in veterinary medicine is still emerging. Similar approaches have demonstrated the ability to use social media analysis to capture the sentiment of backyard poultry owners during the most recent vND outbreak (2018–2020) centered in Southern California (17).

When considered in the context of a foreign animal disease like ND in a complex/diverse urban area such as Southern California, the ability to use these types of data can be utilized to better understand the potential risks associated with pigeon racing and disease spread in Southern California. These types of results in turn can then be used for active response during future outbreaks. Here we used publicly available pigeon related posts on the CAW to understand the spatio-temporal trading activities associated with pigeon racing in Southern California. In summary, the combination of both the above techniques (surveys and web-scraping) was used to better understand the overall scope of racing pigeons in Southern California.

## Methods

### Survey

A survey in English and Spanish was developed using QualtricsTM in collaboration with the California Department of Food and Agriculture (CDFA) and members of a local pigeon racing club (survey in the Appendix). The survey consisted of 24 closed and open-ended questions. The questions were categorized into the following 5 blocks: Block 1: Racing Logistics, Block 2: Housing, Block 3: Husbandry, Block 4: Biosecurity, Block 5: Vaccines. The developed survey was submitted to the Institutional Review Board (IRB) at UC Davis where it was determined that the survey was “exempt” from IRB approval. Once the survey was finalized and approved, surveys were made available via paper or digitally between February 22, 2021 to May 31, 2021. A CDFA member distributed English and Spanish surveys to three bird feed stores frequently visited by racing pigeon owners in Anaheim, Compton and Pomona California. English and Spanish surveys were also distributed via email by the CDFA to 30 pigeon racing club members’ who were known by the CDFA.

### Classified advertisement website monitoring

The CAW was monitored for posts selling birds and/or eggs each day during a 41-day period from 12/21/2020 to 1/31/. Posts located in the Los Angeles County, San Diego County, Inland Empire, Imperial County and Modesto regions were monitored. Posts were Collected if the title contained one or more of the following: “chicken, rooster, hen, bird, pigeon, egg, quail, duck, goose, chick, pullet, gallos, gallina, huevo, pato, pavo, guajolote, codorniz, turkey, aseel, asil, scandaroon, rhode.” If the title contained an item or items from the previous list and also included one or more of “cage, wire, coop, “trailer,” it was ignored.

A web scraper program written in C# was created for this study (18). The main post page of the CAW listing all of the advertisements for each region was provided as an input to the program, and the ScrapySharp library was used to navigate to the website (19). Using the HtmlAgilityPack library, the html for the title text of each listing was identified and matched to the aforementioned search terms using regular expressions (20). URLs for posts matching the study criteria were added to a list. Each individual post page was then opened, and the he titles, description, URL, post-date, collection date, and city or region of the post were recorded in Excel. The web scraper was then directed to navigate to the next page of the CAW main post page, and the process was repeated until all of the listings on the CAW site that day had been examined. To reduce the burden of increased traffic to the monitored CAW, the web scraper was paused for 250 milliseconds between each new http request (i.e., between each programmatic “click” of a link). The number of days since the post was first made was calculated at collection. Since this data was collected as part of a larger study, tags were applied to each post.

According to the contents of the title and description to separate posts by subject. Five possible tags were created: “Egg,” “Chicken,” “Pigeon,” “Fighting,” and “Other Species.” The criteria for each tag was as follows: “Egg” – “egg,” “fertilized,” “dozen,” “huevo”; “Chicken” –“chicken,” “rooster,” “hen,” “pullet,” “gallina”; “Pigeon” – “pigeon”; “Fighting” – “rooster,” “cockerel,” “gallos”; “Other Species” –“quail,” “duck,” “goose,” “pavo,” “pato,” “guajolote,” “turkey,” “codorniz.” If any of the criteria for a tag were present in either the title or description of a post, then the tag was applied. Once collected, the data was combined into a single file and cleaned using the Pandas package in Python (21). Posts that met collection criteria but contained a description selling an unrelated item were removed manually. Since posts may have been online for multiple days and collected multiple times, only posts with a unique universal resource.

Locator (URL) were kept and duplicates were removed using a pivot table in Excel. Once cleaned, the posts tagged “Pigeon” were tagged as “Racing” if the “Title” or “Description” contained any of the following – “race,” “racing,” “racer,” “homer,” “homing” using Python. From the cleaned dataset, the number of posts in each location regardless of tags were counted and mapped using ArcGIS Pro 2.8 based on location. Data for posts containing each tag were separated and mapped based on location using ArcGIS Pro 2.8.

## Results

### Survey

A total of one response from one of the pigeon racing club organizers was received. The response came from a paper survey that was scanned and emailed to the CDFA. Information provided from the one response covered several topics associated with pigeon racing. Spatio-temporally, pigeon racing events occur during the winter and spring, start in Desert Center in Riverside County east of Los Angeles and are typically 240–724 kilometers in length. With respect to husbandry the pigeons are housed out-doors in a fully enclosed area. While there is fencing between each pigeon in the loft, the respondent said that pigeons from different lofts share housing when they are going to an event and that pigeons are allowed to fly up to 1-h on non-racing days. Finally, with respect to biosecurity the respondent stated that they do not share equipment (including carpools to racing events) with other pigeon racers.

### Classified advertisement website monitoring

**Figure 1:**
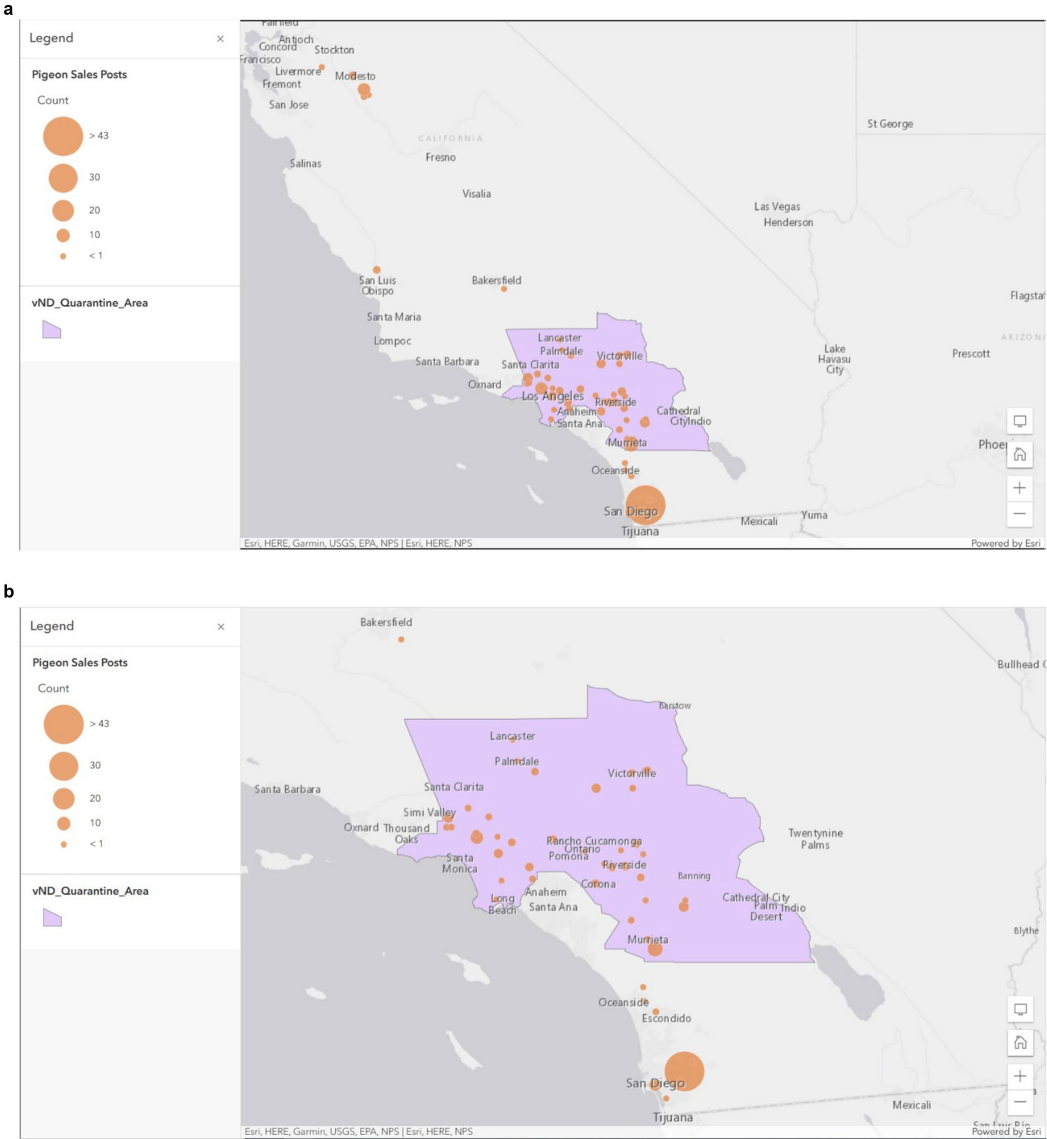
Proportional symbology of unique classified advertisement sales post at the state level **(A)** and within Southern California **(B)** where the vND quarantine zone existed. The orange dots indicate the amount of pigeon posts on the CAW in each city during the time period when sales data was monitored. The pink background represents the vND quarantine area.

**Figure 2:**
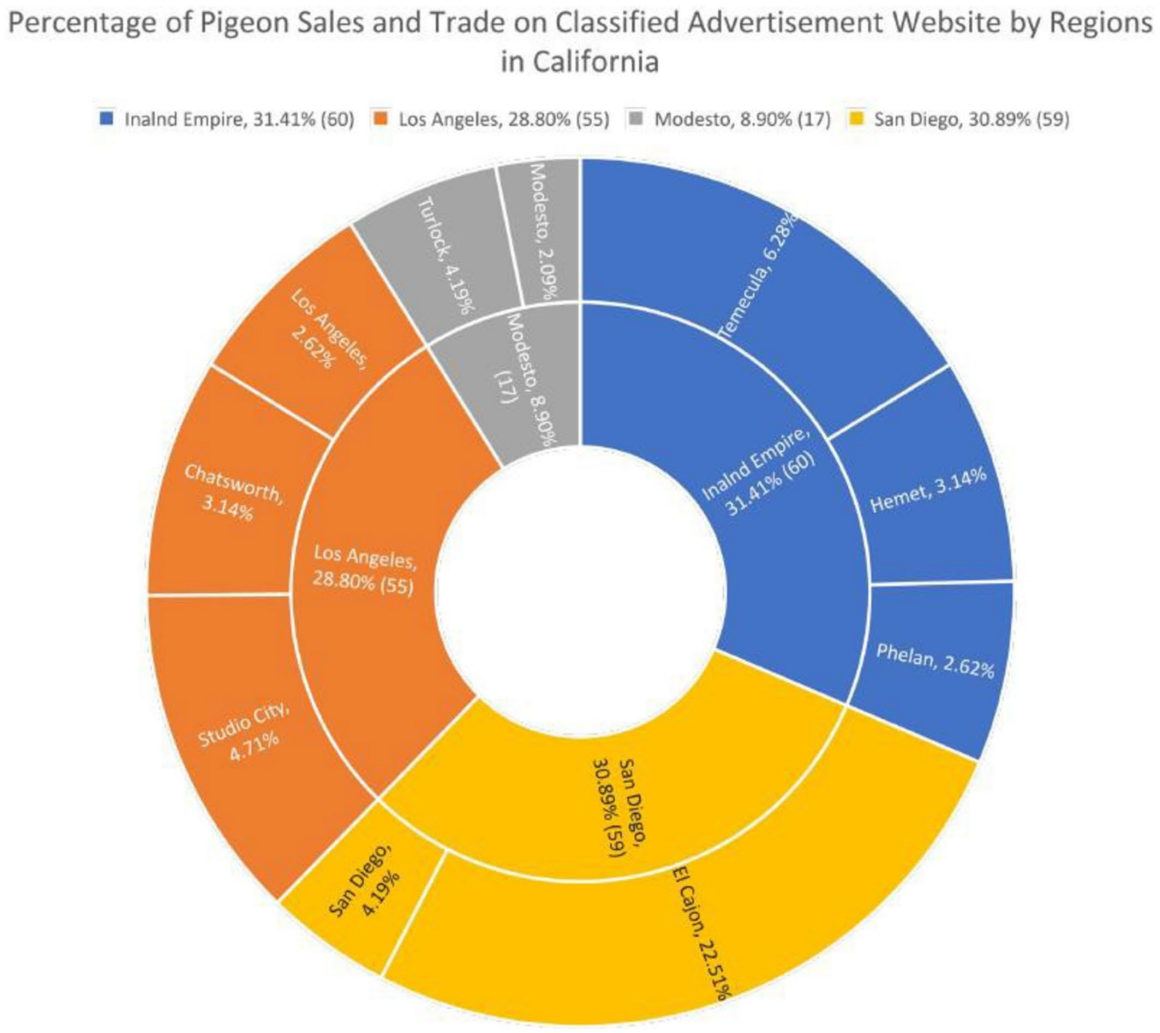
Inner pie chart displays the percentage and total number of pigeon sales and trade from unique posts on the classified advertisement website from 12/21/2020 to 1/21/2021 in San Diego County, Inland Empire region, Los Angeles County, and Modesto area. No pigeons were posted for sale in Imperial County Within each of the remaining regions, the cities with the highest percentage of unique pigeon posts were added into the outer pie chart to locate the exact areas with the most pigeon activity.

Figure 1 shows CAW sales at the state level between 12/21/2020 to 1/21/2021. A total of 191 unique pigeon related posts were observed during the study period. Of these 191 posts, 51 (26.70%) specifically mentioned racing or homing in the “Title” or “Description” of the post. The majority of the web-scraped posts came from Southern California and some posts from the Modesto area (Figure 1a). No pigeons were posted for sale in Imperial County. Figure 1b shows a more detailed view the vND quarantine area overlapped with CAW posts in Southern California (Figure 1b). With respect to specific locations where sales occurred, El Cajon (San Diego County) had 22.51% of all the pigeon posts and represented the highest activity of racing pigeon sales and trade (Figure 2).

To better represent sales at the regional and city level a double pie-chart was generated (Figure 2) with the inner pie delineating the highest number of CAW posts by region as defined by CAW and the outer pie representing the cities with the highest number of posts from associated cities within each of those 4 regions. Within the 4 regions, the Inland Empire region in Southern California, had 31.41% of the unique pigeon posts that were monitored on CL within the 4-week period, which represented 60 unique posts. San Diego County had 30.89% of the observed pigeon posts within the 4-week period, which represented 59 unique pigeon related posts. The remainder of the web scraped pigeon posts on Craigslist came from locations in Los Angeles County (28.80%, *n* = 55) and the Modesto area (8.90%, *n* = 17) respectively.

## Discussion

### Survey

Over a one-month period of time we were able to web-scrape 191 posts on the CA versus only one response via the traditional IRB approved survey. From an extension perspective this demonstrates the potential of web-scraping of social media as a complementary tool to better understand various groups that are traditionally challenging to reach out to such as the pigeon racing industry in Sothern California. While the single response to the survey represents a poor response rate, poor participation rates for surveys are a well-documented challenge with traditional surveys (22–24). This reality combined with anecdotal information provided about the pigeon racing industry in Southern California with respect to privacy and general unfamiliarity with technology (e.g., digital based surveys sent via Qualtrics) are all possible explanations for the poor survey participation rate. More specifically and also important to note, all three feed stores mentioned that the racing pigeon owners were hesitant to fill out the survey for a number of reasons including: the racing pigeon owners are frustrated at the number of times they have had to cancel their races altogether due to an outbreak of ND in Southern California, they believe that ND is a “Chicken”virus and not a pigeon virus, and they also fear losing their pigeons if they work with any.

Regulatory organization. While better outreach with respect to science-based communication has been shown to have a significant impact on knowledge (25), it is likely that even in a “perfect world situation” participation rates will likely remain low. A search of the literature found no surveys of the pigeon racing community. However, even though the participation rate in the survey was low some valuable information was gained in that the survey respondent was head of one of the active racing clubs. Hence the results likely provide some useful information that can be used to better understand the pigeon racing industry in Southern California. For example, understanding the seasonality of the races in addition to one of the starting locations in Southern California can help with identifying potential racing paths (as noted in the introduction the “finish line” is based on the home-location) the pigeons take. Specifically, by using the starting location identified in the survey in combination with the cities with the highest racing pigeon sales identified via web-scraping of CL, spatio-temporal mapping could be used to better inform the commercial poultry industry and the racing pigeon industry about potential threats with respect to biosecurity, disease exposure and transmission.

With respect to surveys it should be noted that while they are the commonly used by extension-based researchers in non-commercial animal agriculture, surveys are typically convenience-based surveys that are not generalizable. That reality combined with the low absolute response rate and low participation rates suggest that new approaches need to be considered especially for groups that are historically difficult to survey.

### Classified advertisement website monitoring

In the United States, over 90% of the population uses the internet and 72.3% use social Media (26, 27) With a large proportion of the population using social media, utilizing web data to understand population level trends including spatio-temporal trends in wildlife trade and visitation data to conservation sites (28). From an extension perspective these types of data could be used to better understand the spatio-temporal dynamics of non-commercial poultry trading activities which could be used to better inform stakeholders focused on outreach, extension and response to various outbreaks of disease (17). This paper provides one of the first examples of how agricultural-based extension professionals can utilize web-scraping as a complimentary approach to traditional surveys. For this study, in contrast to the survey data, monitoring the classified ad website for racing pigeon sales over a relatively short period of time (41 days), resulted in 191 unique web-scraped results, which provided a novel spatio-temporal (Figures 1, 2) tool for identifying the general (at the city level) locations of sales. Since the majority of these sales were located in geographic areas with the vND quarantine zone (Figure 1b) these data suggest that racing pigeons and their associated activities (i.e., racing, husbandry) represent a potentially significant source of disease transmission. While the website was not monitored for long enough to justify temporal analysis, a longer study could be used to identify temporal trends which would add further utility to these types of data as well as more thorough inclusion of multiple languages.

The web data monitoring in this study focused on a single data source in a limited geographic area but scaling up the geographic area and including multiple sources of sales information, including other online marketplaces, brick and mortar stores, mail order catalogues, and open-air markets would provide a more complete understanding of the movements and spatial densities of species of concern. When scaling up the monitoring of web data, the number of data points can become overwhelmingly large for periodic collection by human researchers.

Techniques and tools like web crawling, web scraping, and Application Programing Interfaces (API) based connections, especially in the case of social media marketplaces, make large scale monitoring of these data feasible. When employing web scrapers for such a task, it is important to consider the legal and ethical issues as discussed by (29–31). While the analysis of web-based data including social media will continue to grow, it should be noted that web-scraping is different than simply downloading data as those data are further analyzed for information such as opinion and location. Interestingly, there is no current legislation addressing web-scraping (31). Unlike surveys, web-scraping does not require IRB approval. As web-scraping analysis becomes more powerful and automated guidelines will likely emerge. For this study,

The data collected in this study was publicly available without authentication and could have been collected by a team monitoring the CAW site. Steps were also taken to ensure no large spikes in web traffic burdened the CAW site due to the web scraper program, and all collected data was deidentified. Whenever possible, API connections opened by the owner of a website being monitored should be used.

While the dates that web data was collected from the CAW (12/21/2020–1/31/2021) did not temporally overlap with the vND outbreak (5/2018–2/2020), understanding how various non-commercial avian stakeholders utilize CAW based websites is valuable for developing novel methods of spatio-temporal surveillance. Specifically, web data offers a relatively novel and robust approach toward gaining valuable continuous spatio-temporal data that can be used to better understand disease transmission with respect to racing pigeons in Southern California, the value of traditional surveys as a complementary tool should not be discounted as survey allow for specific questions to be asked. Ultimately, a combination of traditional surveys, web-scraping and associated analysis of those data (e.g., natural language processing, sentiment analysis, spatio-temporal mapping) will facilitate information gathering for effective extension-based efforts. In this use case, the web-scraping methodology described could provide a new layer of useful information for preparing for future outbreaks with a focus on using the spatio-temporal data for disease modeling.

Furthermore, continuous utilization of web data could help identify spatio-temporal trends within the Southern California pigeon racing community that surveys are likely not capable of identifying. From a generic extension level, the analysis of social media can allow for continuous insights about a variety of relevant topics that extension-based professionals work in. This use case represents an initial attempt to utilize this approach.

## Data Availability

The raw data supporting the conclusions of this article will be made available by the authors, without undue reservation.
