# Supplementary material for: Comparing traditional surveys and web-scraped data to understand the pigeon racing industry in Southern California
Source: Front Vet Sci. 2025 Feb 3;11:1451198. doi: 10.3389/fvets.2024.1451198 (PMC11831698; doi:10.3389/fvets.2024.1451198)
Supplement: Supplementary file 1 [file Table_1.docx]

APPENDIX

**Racing and Roller Pigeon Survey:**

Introduction

Thank you for taking the time to participate in this voluntary short survey. The UC Davis

School of Veterinary Medicine-Cooperative Extension and the California Department of

Food and Agriculture (CDFA) will use the information collected to better understand the

risk of disease transmission between backyard poultry and racing/roller pigeons. All

answers you provide are confidential and will only be used for outreach or informational

purposes.

We appreciate your cooperation and trust. If you have any questions/comments please

contact Dr. Maurice Pitesky at mepitesky@ucdavis.edu or Malekah Isa

(maisa@ucdavis.edu). By continuing you indicate that you are of 18 years of age or

older, have read the description of the survey and agree to participate in the voluntary

survey.

Agree

Block 1: Racing Logistics

1. What seasons of the year are your pigeons racing? (select all that apply)

Spring

Summer

Fall

Winter

2. In what regions of California do you take your pigeons to race? (select all that apply)

Greater Sacramento

Bay Area

San Joaquin Valley

Southern California

Northern California

3. In which cities do the races typically start? (Please state)

4. Typically how many pigeons participate in each race?

less than 50 pigeons

50-100 pigeons

100-200 pigeons

200-500 pigeons

500-1000 pigeons

5. Approximately how many miles are the pigeons flying during a race?( Please state)

Block 2: Housing

6. Describe how the birds are housed at your house/ property. (Check all that apply)

Inside the home

outdoors-open top pen or enclosure

outdoors-fully enclosed

individually tethered

free range/loose

7. Do you house or keep birds anywhere else?

Yes

No

Sometimes

8. Do you house other birds for anyone else, for business or as a favor?

Yes

No

Sometimes

9. In the past 30 days, have any new birds been brought onto your property?

Yes

No

If yes, where did they come from? (Please state)

10. Do your pigeons and other pigeons from different lofts share housing when they are

going to events?

Yes

No

Sometimes

11. Do you have fencing between each pigeon in your pigeon loft?

Yes

No

Sometimes

12. Do you travel or carpool with other birds that are not yours to pigeon races?

Yes

No

Sometimes

Block 3: Husbandry

13. Do you breed your own pigeons?

Yes

No

14. Where do you purchase your pigeons? (Check all that apply)

In California

Out of State (Please state where)__________

Out of Country (Please state where)________

15. Please list the name(s) and location(s) of the store(s) where you purchase feed and supplies for your birds.

16. Do you share equipment/feed/supplies with other bird owners?

Yes

No

Sometimes

Block 4: Biosecurity

17. Do you keep other birds on your property?

Yes

No

If so, tell us about the type and number of birds kept on your property. (Please state)

For example,

Backyard Poultry Male/female, #adults, #young, total

Exhibition Birds/Game fowl Male/female, #adults, #young, total

Ducks/Geese Male/female, #adults, #young, total

18. Do your neighbors have birds?

Yes

No

If yes, what type of birds? (Please state)

19. Do you let your pigeons out of their loft when they are not racing?

Yes

No

20. Have any other birds been noticed around your home/property in the past month?

Yes

No

If yes, select that apply

Wild birds co-mingling with your birds (direct contact)

Wild birds near your caged birds (barrier separation)

Sick or dead birds on your property

Poultry on your property

21. Do you clean your pigeon loft?

Yes

No

If yes, how often do you clean your pigeon loft?

twice a day

once a day

once a week

once every two weeks

once a month

Block 5: Vaccines

22. Which vaccines do you use to vaccinate your pigeons?

Pigeon Paramyxovirus (PMV) vaccine

virulent Newcastle Disease vaccine

Other (please state)_______

23. How often do you vaccinate your pigeons?

once a month

every 6 months

every year

every 5 years

once in their lifetime

24. In the past year, have any poultry at your home been vaccinated for Newcastle disease?

Yes

No
